# (3*Z*)-3-[(*Z*)-2-(2-Oxoindolin-3-yl­idene)hydrazin-1-yl­idene]indolin-2-one 0.17-hydrate

**DOI:** 10.1107/S1600536814011805

**Published:** 2014-05-31

**Authors:** Yong-Hong Liu, Lei Zhao, Ming-Xuan Liu, Hai Lin, Jing-Jing Li

**Affiliations:** a College of Chemistry and Chemical Engineering, Yangzhou University, Jiangsu Provincial Key Laboratory of Environmental Material & Engineering, Yangzhou, 225002, People’s Republic of China; b College of Chemistry and Chemical Engineering, Yangzhou University, Yangzhou 225002, People’s Republic of China

## Abstract

In the title compound, C_16_H_10_N_4_O_2_·0.17H_2_O, prepared by the one-step condensation reaction of isatin with hydrazine hydrate under microwave irradiation, the complete organic mol­ecule is generated by crystallographic inversion symmetry and therefore exists in an *S*-*trans* conformation. In the crystal, mol­ecules are linked by N—H⋯O hydrogen bonds, generating a three-dimensional framework with [001] channels, which are occupied by the disordered water mol­ecules.

## Related literature   

For background to microwave synthesis, see: Hoz *et al.* (2004 ▶); Jagani *et al.* (2012 ▶). For our previous work in this area, see: Liu *et al.* (2008 ▶); Wang *et al.* (2010 ▶). For the coventional synthesis of the title compound, see: Ali & Alam (1994 ▶).
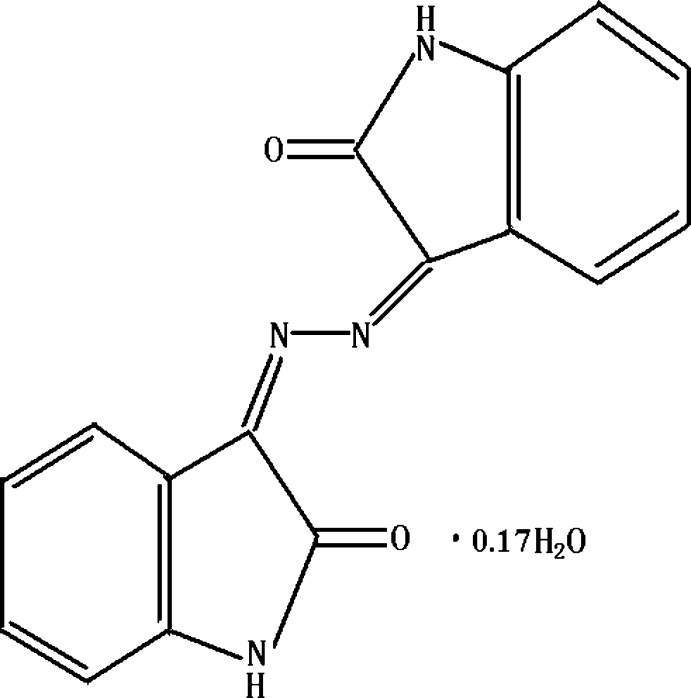


## Experimental   

### 

#### Crystal data  

C_16_H_10_N_4_O_2_·0.17H_2_O
*M* *_r_* = 308.30
Trigonal, 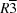

*a* = 24.8699 (18) Å
*c* = 5.6603 (8) Å
*V* = 3031.9 (5) Å^3^
*Z* = 9
Mo *K*α radiation
μ = 0.10 mm^−1^
*T* = 296 K
0.38 × 0.16 × 0.14 mm

#### Data collection  

Bruker SMART1000 CCD diffractometer
Absorption correction: multi-scan (*SADABS*; Bruker, 2002 ▶) *T*_min_ = 0.963, *T*_max_ = 0.986
8691 measured reflections
1547 independent reflections
1290 reflections with *I* > 2σigma(*I*)
*R*_int_ = 0.025

#### Refinement  

*R*[*F* ^2^ > 2σ(*F* ^2^)] = 0.039
*wR*(*F* ^2^) = 0.108
*S* = 1.01
1547 reflections
103 parameters
H-atom parameters constrained
Δρ_max_ = 0.25 e Å^−3^
Δρ_min_ = −0.18 e Å^−3^

### 

Data collection: *SMART* (Bruker, 2002 ▶); cell refinement: *SAINT* (Bruker, 2002 ▶); data reduction: *SAINT*; program(s) used to solve structure: *SHELXS97* (Sheldrick, 2008 ▶); program(s) used to refine structure: *SHELXL97* (Sheldrick, 2008 ▶); molecular graphics: *PLATON* (Spek, 2009) ▶; software used to prepare material for publication: *SHELXTL* (Sheldrick, 2008 ▶).

## Supplementary Material

Crystal structure: contains datablock(s) I, New_Global_Publ_Block. DOI: 10.1107/S1600536814011805/hb7227sup1.cif

Structure factors: contains datablock(s) I. DOI: 10.1107/S1600536814011805/hb7227Isup2.hkl

Supporting information file. DOI: 10.1107/S1600536814011805/hb7227Isup3.cml

CCDC reference: 1004532

Additional supporting information:  crystallographic information; 3D view; checkCIF report

## Figures and Tables

**Table 1:** Hydrogen-bond geometry (Å, °)

*D*—H⋯*A*	*D*—H	H⋯*A*	*D*⋯*A*	*D*—H⋯*A*
N1—H1⋯O1^i^	0.86	2.13	2.8951 (17)	148

Symmetry code: (i) 
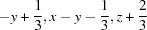
.

**Fig. 1.**
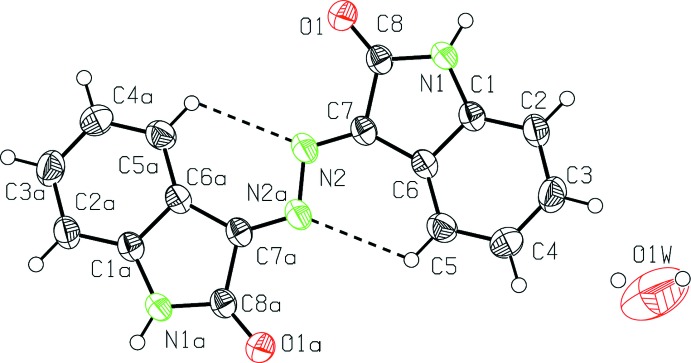
The molecular structure of the title compound, showing 30% probability ellipsoids.

**Fig. 2.**
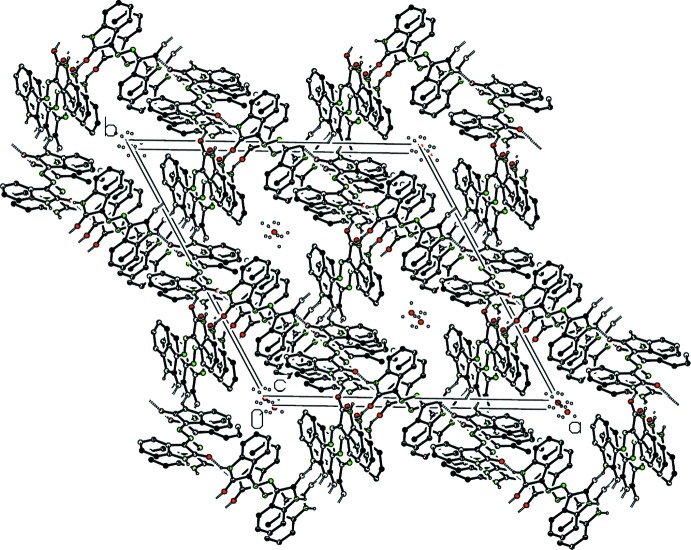
Part of the crystal structure of the title compound, showing two couples of N–H···O inter-molecular hydrogen bonds as dashed lines linking the molecules and disorder water molecules into a super-molecular net-work structure. For the sake of clarity, H atoms not involved in hydrogen bonding have been omitted.

## supplementary crystallographic information

### 1. Comment

Microwave irradiated and solvent-free synthesis have aroused great attention in recent years due to rapid, convenient, green, environment friendly, inexpensive and efficient (Hoz *et al.*, 2004; Jagani *et al.*, 2012). As a continuation of our research work on Schiff bases (Liu *et al.*, 2008; Wang *et al.*,2010), we report here one step synthesis of the title compound under microwave irradiated and free-solvent condition, which was prepared by two steps in the normal method (Ali & Alam, 1994), and its structure.

In the central symmetric molecule of the compound, the non-hydrogen atoms are conjugated by a couple of double bonds of C7=N2 and C7a=N2a, because whose bond length [1.2891 (17) Å] is shorter than the single bond one of C1—N1or C1a—N1a [1.4022 (17) Å] but longer than normal double one of C=N [1.271 (5) Å]. The molecule exists as the most stable configuration of (*E, E*)-isomer and conformation of *s-trans* (Fig. 1, Table 1).

In its pack structure there are two couples of N1–H1···O1 inter-molecular hydrogen bonds in the neighbor molecules which link many molecules into three dimensional net-work frames, and the disorder water molecules merge into the net-work (Fig. 2, Table 1). Thus the guest molecules of the water and the host molecules of the compound form into a super-molecular net-work structure.

### 2. Experimental

In refluxing equipment, isatin (2.94 g, 20 mmol), 50% hydrazine hydrate (0.62 ml, 9.5 mmol) were heated under microwave irradiation for 10 min. After cooling, the red crystalline mixture was recrystallized from dimethylformamide to give 2.5 g (86.2%) of the title compound, m.p. 494.5–495.5 K (ref. 494.5~495.5 K, Ali *et al.*, 1994).

### 3. Refinement

After their location in a difference map, all H atoms were fixed geometrically at ideal positions and allowed to ride on the parent C atoms, with C — H distances of 0.93 (aromaticl CH), O — H distances of 0.84 and N— H distances of 0.86, and with *U_iso_*(H) values of 1.2*U_eq_*(C).

### Crystal data

**Table:**

C_16_H_10_N_4_O_2_·0.17H_2_O	*F*(000) = 1369
*M**_r_* = 308.30	*D*_x_ = 1.450 Mg m^−^^3^
Trigonal, *R*3	Mo *K*α radiation, λ = 0.71073 Å
Hall symbol: -R 3	θ = 2.8–27.2°
*a* = 24.8699 (18) Å	µ = 0.10 mm^−^^1^
*c* = 5.6603 (8) Å	*T* = 296 K
*V* = 3031.9 (5) Å^3^	Block, brown
*Z* = 9	0.38 × 0.16 × 0.14 mm

### Data collection

**Table:**

Bruker SMART1000 CCD diffractometer	1547 independent reflections
Radiation source: fine-focus sealed tube	1290 reflections with *I* > 2σigma(*I*)
Graphite monochromator	*R*_int_ = 0.025
thin–slice ω scans	θ_max_ = 27.5°, θ_min_ = 1.6°
Absorption correction: multi-scan (*SADABS*; Bruker, 2002)	*h* = −32→32
*T*_min_ = 0.963, *T*_max_ = 0.986	*k* = −29→32
8691 measured reflections	*l* = −7→7

### Refinement

**Table:**

Refinement on *F*^2^	Primary atom site location: structure-invariant direct methods
Least-squares matrix: full	Secondary atom site location: difference Fourier map
*R*[*F*^2^ > 2σ(*F*^2^)] = 0.039	Hydrogen site location: inferred from neighbouring sites
*wR*(*F*^2^) = 0.108	H-atom parameters constrained
*S* = 1.01	*w* = 1/[σ^2^(*F*_o_^2^) + (0.0545*P*)^2^ + 2.5765*P*] where *P* = (*F*_o_^2^ + 2*F*_c_^2^)/3
1547 reflections	(Δ/σ)_max_ < 0.001
103 parameters	Δρ_max_ = 0.25 e Å^−^^3^
0 restraints	Δρ_min_ = −0.18 e Å^−^^3^

### Special details

**Table:**

Experimental. The title compound was synthesized under microwave irradiation.
Geometry. All e.s.d.'s (except the e.s.d. in the dihedral angle between two l.s. planes) are estimated using the full covariance matrix. The cell e.s.d.'s are taken into account individually in the estimation of e.s.d.'s in distances, angles and torsion angles; correlations between e.s.d.'s in cell parameters are only used when they are defined by crystal symmetry. An approximate (isotropic) treatment of cell e.s.d.'s is used for estimating e.s.d.'s involving l.s. planes.
Refinement. Refinement of *F*^2^ against ALL reflections. The weighted *R*-factor *wR* and goodness of fit *S* are based on *F*^2^, conventional *R*-factors *R* are based on *F*, with *F* set to zero for negative *F*^2^. The threshold expression of *F*^2^ > σ(*F*^2^) is used only for calculating *R*-factors(gt) *etc*. and is not relevant to the choice of reflections for refinement. *R*-factors based on *F*^2^ are statistically about twice as large as those based on *F*, and *R*- factors based on ALL data will be even larger.

### Fractional atomic coordinates and isotropic or equivalent isotropic displacement parameters (Å^2^)

**Table:**

	*x*	*y*	*z*	*U*_iso_*/*U*_eq_	Occ. (<1)
C1	0.25001 (6)	0.06795 (6)	0.2063 (2)	0.0345 (3)	
C2	0.20279 (7)	0.05119 (7)	0.3672 (3)	0.0429 (3)	
H2	0.1975	0.0250	0.4934	0.051*	
C3	0.16336 (8)	0.07495 (8)	0.3331 (3)	0.0515 (4)	
H3	0.1310	0.0644	0.4390	0.062*	
C4	0.17099 (8)	0.11403 (8)	0.1452 (3)	0.0521 (4)	
H4	0.1434	0.1286	0.1257	0.063*	
C5	0.21929 (7)	0.13147 (7)	−0.0134 (3)	0.0439 (3)	
H5	0.2247	0.1581	−0.1379	0.053*	
C6	0.25943 (6)	0.10844 (6)	0.0170 (2)	0.0341 (3)	
C7	0.31363 (6)	0.11575 (6)	−0.1095 (2)	0.0336 (3)	
C8	0.33439 (6)	0.07495 (6)	0.0163 (2)	0.0349 (3)	
N1	0.29469 (5)	0.04931 (5)	0.20131 (19)	0.0386 (3)	
H1	0.2967	0.0246	0.3026	0.046*	
N2	0.34522 (6)	0.14844 (5)	−0.2881 (2)	0.0406 (3)	
O1	0.37735 (5)	0.06693 (5)	−0.03535 (18)	0.0459 (3)	
O1W	0.0000	0.0000	0.248 (4)	0.177 (8)	0.25

### Atomic displacement parameters (Å^2^)

**Table:**

	*U*^11^	*U*^22^	*U*^33^	*U*^12^	*U*^13^	*U*^23^
C1	0.0402 (7)	0.0309 (6)	0.0306 (6)	0.0163 (5)	0.0026 (5)	0.0005 (5)
C2	0.0495 (8)	0.0409 (7)	0.0372 (7)	0.0217 (6)	0.0112 (6)	0.0069 (6)
C3	0.0532 (9)	0.0532 (9)	0.0516 (9)	0.0291 (8)	0.0181 (7)	0.0037 (7)
C4	0.0582 (9)	0.0549 (9)	0.0574 (10)	0.0388 (8)	0.0097 (7)	0.0033 (7)
C5	0.0547 (9)	0.0410 (7)	0.0422 (8)	0.0287 (7)	0.0055 (6)	0.0060 (6)
C6	0.0412 (7)	0.0297 (6)	0.0297 (6)	0.0165 (5)	0.0034 (5)	0.0017 (5)
C7	0.0385 (7)	0.0309 (6)	0.0281 (6)	0.0149 (5)	0.0011 (5)	0.0005 (5)
C8	0.0394 (7)	0.0345 (6)	0.0292 (6)	0.0173 (5)	0.0018 (5)	0.0003 (5)
N1	0.0461 (6)	0.0415 (6)	0.0327 (6)	0.0252 (5)	0.0067 (5)	0.0103 (5)
N2	0.0460 (7)	0.0416 (6)	0.0335 (6)	0.0214 (5)	0.0066 (5)	0.0095 (5)
O1	0.0479 (6)	0.0568 (6)	0.0415 (6)	0.0325 (5)	0.0074 (4)	0.0050 (5)
O1W	0.093 (6)	0.093 (6)	0.34 (3)	0.047 (3)	0.000	0.000

### Geometric parameters (Å, º)

**Table:**

C1—C2	1.3756 (19)	C5—C6	1.388 (2)
C1—N1	1.4022 (17)	C5—H5	0.9300
C1—C6	1.4073 (17)	C6—C7	1.4554 (18)
C2—C3	1.389 (2)	C7—N2	1.2891 (17)
C2—H2	0.9300	C7—C8	1.5254 (18)
C3—C4	1.388 (2)	C8—O1	1.2165 (17)
C3—H3	0.9300	C8—N1	1.3594 (17)
C4—C5	1.384 (2)	N1—H1	0.8593
C4—H4	0.9300	N2—N2^i^	1.404 (2)
			
C2—C1—N1	127.63 (12)	C6—C5—H5	120.6
C2—C1—C6	122.16 (13)	C5—C6—C1	119.53 (12)
N1—C1—C6	110.21 (11)	C5—C6—C7	134.43 (12)
C1—C2—C3	117.17 (13)	C1—C6—C7	106.04 (11)
C1—C2—H2	121.4	N2—C7—C6	134.34 (12)
C3—C2—H2	121.4	N2—C7—C8	118.92 (12)
C4—C3—C2	121.72 (14)	C6—C7—C8	106.71 (10)
C4—C3—H3	119.1	O1—C8—N1	126.85 (12)
C2—C3—H3	119.1	O1—C8—C7	127.86 (12)
C5—C4—C3	120.64 (14)	N1—C8—C7	105.29 (11)
C5—C4—H4	119.7	C8—N1—C1	111.72 (10)
C3—C4—H4	119.7	C8—N1—H1	124.1
C4—C5—C6	118.75 (13)	C1—N1—H1	124.2
C4—C5—H5	120.6	C7—N2—N2^i^	111.89 (14)
			
N1—C1—C2—C3	−178.92 (14)	C5—C6—C7—C8	−177.99 (15)
C6—C1—C2—C3	1.3 (2)	C1—C6—C7—C8	1.50 (14)
C1—C2—C3—C4	0.0 (2)	N2—C7—C8—O1	−2.8 (2)
C2—C3—C4—C5	−1.1 (3)	C6—C7—C8—O1	178.67 (13)
C3—C4—C5—C6	0.9 (2)	N2—C7—C8—N1	176.85 (12)
C4—C5—C6—C1	0.3 (2)	C6—C7—C8—N1	−1.65 (14)
C4—C5—C6—C7	179.71 (15)	O1—C8—N1—C1	−179.13 (13)
C2—C1—C6—C5	−1.4 (2)	C7—C8—N1—C1	1.18 (14)
N1—C1—C6—C5	178.74 (12)	C2—C1—N1—C8	179.94 (13)
C2—C1—C6—C7	178.98 (12)	C6—C1—N1—C8	−0.26 (15)
N1—C1—C6—C7	−0.83 (14)	C6—C7—N2—N2^i^	0.1 (2)
C5—C6—C7—N2	3.8 (3)	C8—C7—N2—N2^i^	−177.92 (13)
C1—C6—C7—N2	−176.67 (15)		

Symmetry code: (i) −*x*+2/3, −*y*+1/3, −*z*−2/3.

### Hydrogen-bond geometry (Å, º)

**Table:**

*D*—H···*A*	*D*—H	H···*A*	*D*···*A*	*D*—H···*A*
N1—H1···O1^ii^	0.86	2.13	2.8951 (17)	148

Symmetry code: (ii) −*y*+1/3, *x*−*y*−1/3, *z*+2/3.
